# A worldwide epidemiological database for COVID-19 at fine-grained spatial resolution

**DOI:** 10.1038/s41597-022-01245-1

**Published:** 2022-03-29

**Authors:** Emanuele Guidotti

**Affiliations:** University of Neuchâtel, Institute of Financial Analysis, Neuchâtel, 2000 Switzerland

**Keywords:** Viral infection, Computational biology and bioinformatics

## Abstract

This database provides the daily time-series of COVID-19 cases, deaths, recovered people, tests, vaccinations, and hospitalizations, for more than 230 countries, 760 regions, and 12,000 lower-level administrative divisions. The geographical entities are associated with identifiers to match with hydrometeorological, geospatial, and mobility data. The database includes policy measures at the national and, when available, sub-national levels. The data acquisition pipeline is open-source and fully automated. As most governments revise the data retrospectively, the database always updates the complete time-series to mirror the original source. Vintage data, immutable snapshots of the data taken each day, are provided to ensure research reproducibility. The latest data are updated on an hourly basis, and the vintage data are available since April 14, 2020. All the data are available in CSV files or SQLite format. By unifying the access to the data, this work makes it possible to study the pandemic on a global scale with high resolution, taking into account within-country variations, nonpharmaceutical interventions, and environmental and exogenous variables.

## Abstract (summary)

**Table:** 

Measurement(s)	cases • deaths • recovered • tests • vaccines • hospitalizations • population
Technology Type(s)	digital curation • digital curation
Sample Characteristic – Organism	coronaviridae
Sample Characteristic – Location	global

## Background & Summary

Since the outbreak of the disease, governments across the globe have been releasing data to track the COVID-19 pandemic as it unfolds. However, the lack of international standards has led to various datasets in local languages and different formats, ranging from highly structured data (e.g., application programming interfaces) to unstructured data (e.g., daily tweets by the national ministry of health). This makes international comparisons of large, detailed outbreak data difficult and prevents inferences from such data from being effective in response to the disease outbreak^1^.

Several collective actions have been taken to harmonize the amount of heterogeneous data available^2^. In particular, the Center for Systems Science and Engineering at the Whiting School of Engineering, with technical support from ESRI and the Johns Hopkins University (JHU) Applied Physics Laboratory, has become the gold standard for national cases and death counts^3^. Similarly, Our World in Data has put together a cross-country database of COVID-19 testing^4^ and a global database of COVID-19 vaccinations^5^ at the national level.

However, national counts only represent less than 2% of the available governmental data (Table 1). Moreover, reporting national statistics ignores significant within-country variation in the disease dynamics^6^. Comparing data between countries also has some limitations due to differences in the reporting criteria^7^. Although only the creation of international standards would fully overcome cross-country comparability issues, a fine-grained database mitigates the limitation of national-level datasets and enables researchers to exploit within-country variations while controlling for country fixed effects.

**Table 1:** The table reports the epidemiological variables included in the database and their coverage as of November 27, 2021.

Field	Description	N. of Administrative Areas (N. of Countries)		
		Level 1	Level 2	Level 3
confirmed	Cumulative number of confirmed cases	226 (226)	759 (33)	12,119 (16)
deaths	Cumulative number of deaths	214 (214)	672 (29)	11,657 (12)
recovered	Cumulative number of recovered people	206 (206)	482 (21)	1,762 (5)
tests	Cumulative number of tests	139 (139)	382 (19)	686 (6)
vaccines	Cumulative number of total doses administered	223 (223)	305 (16)	6,837 (7)
people_vaccinated	Cumulative number of people who received at least one vaccine dose	223 (223)	310 (17)	11,269 (10)
people_fully_vaccinated	Cumulative number of people who received all doses prescribed by the vaccination protocol	223 (223)	310 (17)	11,269 (10)
hosp	Number of hospitalized patients on date	43 (43)	195 (10)	164 (3)
icu	Number of hospitalized patients in intensive therapy on date	39 (39)	202 (9)	164 (3)
vent	Number of patients in intensive therapy requiring invasive ventilation on date	8 (8)	62 (5)	11 (1)

The last three columns report the number of countries (level 1), sub-national regions (level 2), and lower-level administrative areas (level 3) for which the corresponding variable is available. The number of unique countries is given in parentheses.

This paper presents a unified database harmonizing open governmental data around the globe at fine-grained spatial resolution. The project started in March 2020, with the implementation of an extendable package for the R statistical environment^8^ designed to aggregate the data from several sources^9^. This paper presents the data and the infrastructure matured in almost two years of development and maintenance.

**Fig. 1:**
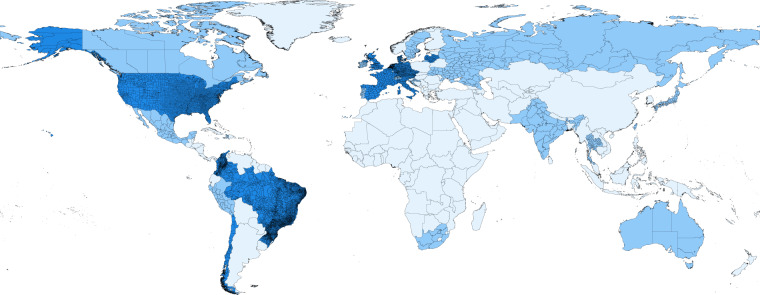
The figure displays the spatial coverage and the granularity of the database as of November 27, 2021. In light blue: countries for which only national-level data are available. In blue: regions for which sub-national data are available. In dark blue: lower level areas for which finer-grained data are available.

At the time of writing, the database provides the time-series of the number of confirmed cases, deaths, recovered people, tests, vaccinations, and hospitalizations, for more than 230 countries, 760 regions, and 12,000 lower-level administrative divisions (see Fig. 1). The description and the coverage for each variable are reported in Table 1. The database includes policy measures at national and, when available, sub-national levels, by Oxford COVID-19 Government Response Tracker^10^. The geographical entities are associated with a set of identifiers that allow merging the epidemiological data with Google mobility reports (https://www.google.com/covid19/mobility), with Apple mobility trends (https://covid19.apple.com/mobility), with the Hydromet dataset (https://github.com/CSSEGISandData/COVID-19_Unified-Dataset), and with spatial databases such as Eurostat (https://ec.europa.eu/eurostat/web/gisco) for Europe or GADM (https://www.gadm.org) worldwide.

The data acquisition pipeline is fully automated. As most governments revise the data retrospectively, the database always updates the complete time-series to mirror the original provider. Vintage data, immutable snapshots of the data taken each day, are provided to ensure research reproducibility. The latest data are updated on an hourly basis, and the vintage data are available since April 14, 2020. All the data are provided in CSV files or SQLite format, both hosted on a cloud storage and freely available to the general public. All the code is open-source.

The database is being used by researchers throughout the pandemic to investigate the relationship between the pandemic and crime^11^, to study sub-national dynamics^6^, to empirically assess government policies^12^ and human movement restrictions^13^, to evaluate the application of machine learning in exploring determinant factors of the pandemic^14^, to detect climatic signatures in the different waves across both hemispheres^15^, and for a variety of other cross-sectional and time-series studies^16–22^. By unifying the access to the data, this work makes it possible to study the pandemic on its global scale with high resolution, taking into account within-country variations, nonpharmaceutical interventions, and environmental and exogenous variables.

## Methods

The basic idea is mapping fine-grained worldwide epidemiological data into a standardized table consisting of rows and columns. Each row represents an observation for a geographical entity at a given point in time, and each column represents an epidemiological variable. The value in each cell is the value of the corresponding variable for the geographical entity at the given point in time. This format is commonly known as tidy data^23^.

Several challenges must be considered. First, it is unclear which identifier should be used for geographical entities, as no standard exists on a global scale with fine-grained resolution. Second, the geographical entities are typically referenced in different ways by different sources. For instance, Italy provides vaccination data where each region is identified using the Nomenclature of Territorial Units for Statistics (NUTS) defined by the European Commission, while confirmed cases are provided in a different governmental dataset that uses the region’s name. The data can be merged only after a link is established between the different identification systems. Third, most governments are revising the data retrospectively. This implies that incremental updates of the database would be faulty. Finally, for such a database to be useful, it should be possible to merge the epidemiological data with exogenous indicators and geospatial information.

The adopted solution consists of a software for the R statistical environment, organized in three building blocks:

- **Data sources**: each data source corresponds to an R function. The input of the function is the level of granularity of the data, i.e., 1 for national-level data, 2 for sub-national data, and 3 for lower-level data. The output of the function is a standardized data frame containing (a subset of) the variables in Table 1. The function downloads the data from the provider for the given level of granularity and maps them into the standardized tidy data format. Each source uses a different identification system for the geographical entities at this stage.
- **Lookup tables**: these are CSV files containing the mapping between several identification systems. Each row represents a geographical entity, and it is identified by a unique code generated by a hash function. The hash code is associated with the various identifiers used by different data sources for the given geographical entity. A second set of covariates is also reported (see Table 2). The two sets are located in the same table for convenience but they play a different role from a conceptual perspective. The first set of identifiers allows merging the epidemiological data provided by different data sources. This is done entirely within the software, and these identifiers are not exposed to the end-user. The second set of identifiers plays no role in the data aggregation pipeline but it is provided to the end-user and allows the merging of the epidemiological data with external databases.

**Table 2:** The table reports the covariates, other than epidemiological variables, included in the database and their coverage as of November 27, 2021.

Field	Description	N. of Administrative Areas		
		Level 1	Level 2	Level 3
id	Unique identifier for the geographical entity	236	763	12,124
administrative_area_level	Level of the administrative area: 1 for countries; 2 for states, regions, cantons, or local equivalent; 3 for cities, municipalities, or local equivalent	236	763	12,124
administrative_area_level_1	Name of the administrative area of level 1	236	763	12,124
administrative_area_level_2	Name of the administrative area of level 2		763	12,124
administrative_area_level_3	Name of the administrative area of level 3			12,124
latitude	Latitude	229	763	12,124
longitude	Longitude	229	763	12,124
population	Total population	235	763	12,124
iso_alpha_3	3-letter code of the country according to the standard ISO 3166-1 Alpha-3	233	763	12,124
iso_alpha_2	2-letter code of the country according to the standard ISO 3166-1 Alpha-2	233	763	12,124
iso_numeric	Numeric code of the country according to the standard ISO 3166-1 Numeric	232	763	12,124
iso_currency	3-letter code of the currency used in the country according to the standard ISO 4217	233	763	12,124
key_local	The administrative area identifier used by the local authorities, usually the national institute of statistics or local equivalent. E.g., FIPS codes for United States, IBGE codes for Brazil, ISTAT codes for Italy, etc.		568	12,053
key_google_mobility	The place_id used in Google Mobility Reports. The identifier also allows to interact with the Google Places and Google Maps API	135	539	6,825
key_apple_mobility	The administrative area identifier identifier used in Apple Mobility Reports. This is constructed by concatenating region and, when not null, sub-region in Apple Mobility Reports: i.e., <region>, <sub-region>	67	475	2,159
key_jhu_csse	The administrative area identifier identifier used in the JHU CSSE Unified COVID-19 Dataset. In particular, this enables to match administrative areas with the Hydromet dataset	192	508	10,708
key_nuts	The 2021 NUTS codes for Europe, by Eurostat		232	1,864
key_gadm	The identifier (GID) used in the GADM database version 3.6	233	760	11,977

The last three columns report the number of countries (level 1), sub-national regions (level 2), and lower-level administrative areas (level 3) for which the corresponding variable is available.
- **Countries**: each country corresponds to an R function. The function takes as input the level of granularity and calls all the data sources needed for the given country. For instance, sub-national data for the United States are provided by The New York Times (cases and deaths), by the Department of Health and Human Services (tests and hospitalizations), and by the Centers for Disease Control and Prevention (vaccination data). In general, the data retrieved by the different sources use a different identification system. The function reads the lookup tables described above to map the different identifiers into the unique hash codes. Then, it merges all the data from the various sources and returns the result. This is now a standardized data frame containing all the geographical entities for the given country and the desired level of granularity. A unique hash code identifies each entity.

**Fig. 2:**
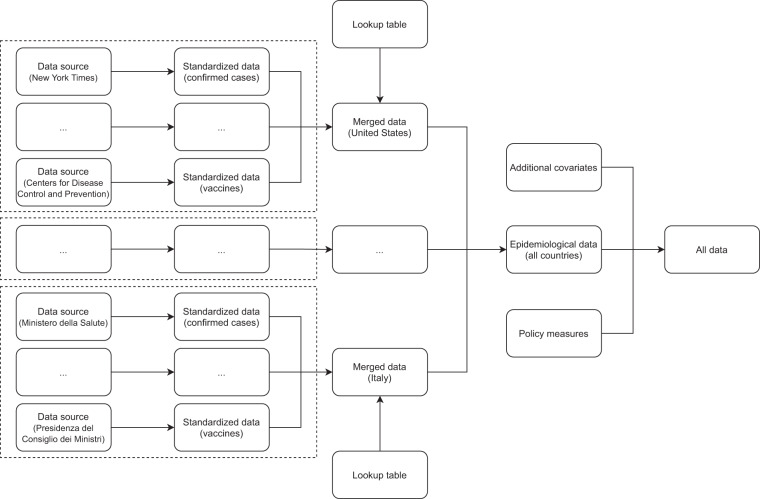
Workflow design. For a given level of granularity and for each country, the epidemiological data are downloaded from several sources, mapped into a standardized data frame, and merged using the lookup tables. Then, a top-level function collects the data for all the countries at the desired level of granularity. Finally, the epidemiological data are merged with additional covariates and with policy measures. Examples of data sources, variables, and countries are given in parentheses.

The workflow is represented in Fig. 2. For a given level of granularity and for each country, the epidemiological data are downloaded from several sources, mapped into a standardized data frame, and merged using the lookup tables. Then, a top-level function collects all the data for all the countries (at the desired level of granularity) and adds the covariates included in the lookup tables and the policy measures by Oxford COVID-19 Government Response Tracker.

The software effectively deals with retrospective updates, as all the data are downloaded from the original provider on the fly. Moreover, its modular design makes it easy to switch data providers whenever needed. However, building the full dataset requires downloading and processing several gigabytes of data and takes between 1-2 hours, even when a high-speed internet connection is used. Therefore, cloud computing is used to address these limitations and simplify access to the data.

All the code is run on a dedicated server as a Linux daemon that runs continuously in the background and automatically re-starts in case of system failure or reboot. The daemon runs the latest version of the software, downloads the data from the providers, and updates a local SQLite database on a continuous basis. As the data live in a persistent storage, sanity checks are implemented before overwriting the data in the database with the latest version from the provider. Then, on an hourly basis, a separate cron job creates a copy of the SQLite database, exports the data in CSV files, and uploads them on a cloud storage available to the general public.

### Data collection

The goal is to mirror the original provider without altering the data. The only operations performed are those strictly necessary to standardize the data, including e.g., computing cumulative cases from daily counts.

Only in very specific cases the data can be aggregated to construct e.g., regional counts from sub-regional counts. In general, aggregating the data bottom-up produces incorrect results due to missing data or cases of unknown origin. Therefore, the data for different levels of granularity are pulled from different sources that directly provide the counts at the desired level.

Non-geographical entities, such as data on repatriated travelers, are dropped. The only exception is a few cruise ships, such as the Diamond Princess. No other cleaning procedure is applied, even in cases where the data may seem incorrect. For instance, the original data provider may report decreasing cumulative counts that lead to negative daily counts (typically due to changes in the data collection methodology). If the provider corrects the data retrospectively, the changes are reflected in the database.

### Data sources

The preferred data sources are open governmental data provided in a machine-readable format. The source must provide the complete time-series and not only the latest counts. However, it is not rare that the official data are scattered in different websites, policy documents, and in a range of unstructured formats.

Alternative data sources are represented by non-official groups collecting official data. The open-source community has been exceptionally active in collating data from unstructured official sources. JHU^3^ and Our World in Data^4,5^ are examples for worldwide data at the national level. Several other repositories, curating specific countries with higher resolution, are also available, typically on GitHub.

To help users to assess the reliability of the data and decide how to use and compare them, information about the sources is provided for each country, level of granularity, and epidemiological variable. Before November 15, 2021, the data sources were listed in CSV files. After that date, the sources are coded in the same script that imports the data, together with the documentation of the software. The documentation is automatically converted in PDF format every time the database updates and it is stored together with the data. The PDF file contains both the reference to the data provider and the link to the script that is used to import the data. This makes it possible to retrieve additional detailed information about the epidemiological variables and to inspect the code that is used to process the original data.

### Lookup tables

The link between different identifiers for the same geographical entity can be established programmatically only in a few cases. The lookup tables are created by first matching the identifiers with a variety of computational techniques, and ultimately inserting the data manually, whenever an exact match could not be found. The name of the administrative division, its population, the corresponding code used by the local authorities, and the identifiers used in external databases are also included in the same way.

### Spatial data

The lookup tables include the identifier (GID) used in the GADM database version 3.6. This is a one-to-one relation as one GID is associated with a unique administrative area. However, it is impossible for some administrative areas to find the exact correspondence in the GADM database. In these cases: (a) when the administrative area includes multiple areas in GADM, the GID of the area closer to the centroid is used; (b) when the administrative area is a subdivision not present in GADM, a new GID is created by appending. n_0 to the GID of the upper division, where n is a sequential integer, and 0 denotes the version number. Latitude and longitude are obtained by downloading geospatial data from GADM and generating a point that lies on the surface of the administrative area.

The 2021 NUTS codes for Europe are added by using geospatial data by Eurostat and by checking which NUTS contains the coordinates computed with GADM. This is a one-to-many relation as one NUTS code can be associated with multiple administrative areas. The lowest level used is NUTS 3. Local Administrative Units (LAU) are mapped to NUTS 3.

### Policy measures

Oxford COVID-19 Government Response Tracker provides policy measures^10^. As the policies often vary within a country, the Tracker reports only the most stringent policy that is in place. The policies have a flag for whether they are *targeted* to a specific geographical region or whether they are a *general* policy that is applied across the whole country.

This database reports *general* policies using a scale of ordinal values, as coded by the Tracker. Instead, *targeted* policies are coded by placing a minus sign in front of their value. The negative sign is used solely to distinguish the two cases; it should not be treated as a negative value.

All the national policies are used for sub-national and lower-level areas unless sub-national policies are directly available from the Tracker. When data on sub-national policies are available, policies at the lowest level are inherited from those at the sub-national level. This implies that positive integers identify policies applied to the entire administrative area. Negative integers are used to identify policies that represent the best guess of the policy in force but may not represent the actual status of the given area.

The database also includes several indices that the Tracker calculates to give an overall impression of government activity. The index values are propagated from upper-level areas to lower-level areas as described for individual policy measures. When the index is not provided directly for the given area, and it is inherited from an upper-level area, a minus sign is placed in front of its value to distinguish the two cases.

## Data Records

All the data are available to download at https://covid19datahub.io. The complete documentation, including the references to the original data sources, is also available at the same link. The data are provided in a single SQLite database or, alternatively, in multiple CSV files. Both formats are updated hourly.

The SQLite database contains all the data and it is organized in two tables. The first table (location) contains information about the geographical entities (see Table 2). The second table (timeseries) contains the time-series of epidemiological variables (see Table 1) and policy measures for each location. The two tables can be merged on the column id.

The CSV files contain the merged data. Each file contains a different subset of the full database. A first option is to download the data by level of granularity. Here, a first file contains worldwide country-level data, a second file contains worldwide sub-national data, and a third file contains worldwide lower-level data. Another option is to download the data for specific countries. Here, one CSV file is provided for each country and contains all the country’s national, sub-national, and lower-level data. A third option is to download the data for single locations, as one CSV file is also provided individually for each location. All the files can be downloaded in a compressed format to save bandwidth and speed up the file transfer.

As most governments revise the data retrospectively, vintage data are provided to simplify the reproducibility of academic research. These are immutable snapshots of the data taken each day since April 14, 2020. The vintage data are provided in SQLite databases and the data sources are listed in PDF files after November 15, 2021. Before this date, the data were provided in ZIP folders and the sources in CSV files, but the file format is generally compatible with the version described in this paper. A copy of the database, as of November 27, 2021, has been uploaded to figshare^24^.

## Technical Validation

The database collects publicly available data published by official sources. As such, the critical quality concern for the database itself is whether it reflects the original providers faithfully. Four main strategies are employed for ensuring this.

First, the data are sample-checked manually when initially integrated into the database. They can be directly checked against the original data files in some cases. In other instances, i.e., for derived data, this is not possible. For example, countries typically provide statistics on the vaccination doses (e.g., number of first, second, one-shot, or booster doses) but may not give directly the number of people vaccinated, which needs to be computed from the doses. In these cases, the final result is checked against governmental websites and dashboards that compute and report this kind of information.

Second, automated tests validate the format of the data and their relations. They check e.g., that dates are in a valid, unambiguous format, that there are no duplicated counts for each location on a given date, that confirmed cases do not exceed the number of tests, that deaths do not exceed the number of cases, that fully vaccinated people are less than the number of people with at least one vaccine dose, and so on. Many issues of this kind are found in the official data, especially at sub-national and lower levels, typically due to differences in reporting criteria or different starting dates used to compute cumulative counts. All the issues are logged and, after manual inspection, notified to the data curator. Although the providers quickly fixed issues due to manual entry mistakes, problems due to different reporting criteria persist. This highlights the need for centralized governmental infrastructures that would help in improving the data quality and consistency.

Third, additional sanity checks are implemented before overwriting the data in the database with the latest version from the provider. This includes e.g., checking that the data in the database are a subset of the latest version from the provider and that the provider is not deleting older data. This strategy avoids corrupted versions of the data and overcomes temporary issues experienced by the data source. Moreover, the database saves the time of the last update to spot out-of-date sources that need a manual inspection.

Finally, all the code is open-source, and the database, downloaded more than 5 million times, is continuously used and monitored by researchers.

## Usage Notes

The database is published under the CC BY license and it is freely available to the general public. The data can be accessed in SQLite (https://sqlite.org), or imported into any software by reading the CSV files described above.

Two packages are made available for R and Python to simplify the interaction with the data and merge them with external databases. The stable release of the R package COVID19 is available on CRAN (https://cran.r-project.org/package=COVID19). The stable release of the Python package covid19dh is available on PyPI (https://pypi.org/project/covid19dh). The development version of both packages is hosted on GitHub (https://github.com/covid19datahub).

## Data Availability

All the code used to generate the database is open-source and available at https://github.com/covid19datahub/COVID19.
